# *ALS3* Expression as an Indicator for *Candida albicans* Biofilm Formation and Drug Resistance

**DOI:** 10.3389/fmicb.2021.655242

**Published:** 2021-04-29

**Authors:** Keke Deng, Wei Jiang, Yanyu Jiang, Qi Deng, Jinzhong Cao, Wenjie Yang, Xuequn Zhao

**Affiliations:** ^1^Department of Respiratory, Tianjin Third Central Hospital Branch, Tianjin, China; ^2^Department of Infectious Disease, Tianjin First Central Hospital, School of Medicine, Nankai University, Tianjin, China; ^3^Department of Hematology, Tianjin First Central Hospital, School of Medicine, Nankai University, Tianjin, China

**Keywords:** *Candida albicans*, biofilms, resistance, gene expression, *ALS3* gene

## Abstract

Resistance caused by the formation of the *Candida albicans* (*C. albicans*) biofilm is one of the main reasons for antifungal therapy failure. Thus, it is important to find indicators that predict *C. albicans* biofilm formation to provide evidence for the early prevention and treatment of the *C. albicans* biofilms. In this study, *C. albicans* samples were selected from *C. albicans* septicemia that were sensitive to common antifungal agents. It was found that the agglutinin-like sequence 3 (*ALS3)* gene was differentially expressed in free, antifungal, drug-sensitive *C. albicans*. The average *ALS3* gene expression was higher in the *C. albicans* strains with biofilm formation than that in the *C. albicans* strains without biofilm formation. Then, it was further confirmed that the rate of biofilm formation was higher in the high *ALS3* gene expression group than that in the low *ALS3* gene expression group. It was found that *C. albicans* with biofilm formation was more resistant to fluconazole, voriconazole, and itraconazole. However, it maintained its sensitivity to caspofungin and micafungin *in vitro* and in mice. Further experiments regarding the prevention of *C. albicans* biofilm formation were performed in mice, in which only caspofungin and micafungin prevented *C. albicans* biofilm formation. These results suggest that the expression level of *ALS3* in *C. albicans* may be used as an indicator to determine whether *C. albicans* will form biofilms. The results also show that the biofilm formation of *C. albicans* remained sensitive to caspofungin and micafungin, which may help to guide the selection of clinical antifungal agents for prevention and therapy.

## Introduction

Infection is one of the most common serious complications associated with the use of medical devices retained in the body, particularly central venous catheters. As a major cause of catheter-related bloodstream infections, *Candida albicans* (*C. albicans*) has the propensity to form biofilms. Compared with its planktonic form, the biofilm formation of *C. albicans* is up to 1,000 times more resistant to azole antifungal agents (Lamfon, 2004) and up to 20 times more resistant to echinocandins (Nett et al., 2010; Tobudic et al., 2010; Taff et al., 2013). Because of its ability to form biofilms, the treatment of *C. albicans* catheter-related infections is challenging (Walraven and Lee, 2013). Recent evidence suggested that even when the minimal inhibitory concentration (MIC50) increases, *C. albicans* biofilm formation remains sensitive to echinocandins (Ramage et al., 2005; Choi et al., 2007; Katragkou et al., 2008).

It is important to identify which *C. albicans* strains have the tendency to form biofilms. A goal of this study was to find some indicators that could predict the biofilm formation of *C. albicans*, which may provide a basis for the early prevention and treatment of *C. albicans* biofilm formation. In previous studies (Deng et al., 2016, 2017), *C. albicans* was resistant to fluconazole, voriconazole, and itraconazole after biofilm formation. The MIC50 of caspofungin and micafungin to 50% of the tested *C. albicans* biofilm increased to different degrees, but they did not reach drug resistance. Further studies found that there were differences in the expression levels of genes in the ALS gene family, particularly *ALS3*, between *C. albicans* strains before biofilm formation. *C. albicans* with higher *ALS3* expression levels tended to form biofilms.

This study selected sensitive *C. albicans* that were isolated from blood samples of *C. albicans* septicemia to investigate the correlation between *ALS3* expression and *C. albicans* biofilm formation and resistance. Then, an *in vivo* experiment to study the prevention of *C. albicans* biofilm formation in mice was performed.

## Materials and Methods

### Source of *Candida albicans* Strains

A total of 55 strains of *C. albicans* isolated from the blood samples of 55 patients with *C. albicans* septicemia who were admitted to the hospital from January 2017 to December 2018 were collected. The patients had not received antifungal therapy before the diagnosis of *C. albicans* septicemia and had not received catheter therapy. All *C. albicans* strains were sensitive to common antifungal agents.

### Strains and Purification

A small number of clinically isolated *C. albicans* was selected via inoculation ring. *C. albicans* were inoculated on Sabouraud dextrose agar (SDA) using a three-zone scribing method and placed into a temperature box at 37°C overnight. The following day, one of the growing strains was transplanted into 5 mL of yeast peptone glucose (YPG) medium and cultured overnight in a shaking table at a speed of 200 rpm at 35°C. On the third day, the medium was removed from the shaker and centrifuged for 5 min at 3,000 rpm to collect the thalli. The obtained thalli were rinsed with saline solution three times and diluted with RPMI-1640 medium. *C. albicans* were then subcultured in yeast peptone and glucose medium at 150 rpm and 35°C in a shaking table overnight (Deng et al., 2016, 2017). The concentration of *C. albicans* solution was adjusted to 1 × 10^7^ cells/mL.

### Drug Sensitivity Test *in vitro*

The susceptibilities of *C. albicans* strains to antifungal agents, including fluconazole (Diflucan, Pfizer Manufacturing Deut.), voriconazole (Vfend, Pfizer Manufacturing Deut.), itraconazole (Sporanox, Xian-Janssen Pharmaceutical Ltd.), caspofungin (Concidas, Merck & Co., Inc.), and micafungin (Mycamine, Astellas Pharma Tech Co., Ltd), were assayed *in vitro* using a microliquid-based dilution method M27-A3 [Clinical and Laboratory Standards Institute (CLSI), 2008]. The ATCC control used in this study was AT0CC10231.

### Expression of *ALS3* in 55 Strains *C. albicans*

To detect *ALS3* expression levels, total RNA extracted from grinded fungi with TRIzol reagent (Invitrogen, Carlsbad, CA, United States) was used as the template for all reverse transcriptase reactions. The cDNA was synthesized with random priming using 10 μL of total RNA and RevertAid First Strand cDNA Synthesis Kit (Fermentas, CA, United States) following the manufacturer’s protocol. The upstream primer of *ALS3* was 5′-CCGGTTTCATCTGAATCATTTAGTT-3′. The downstream primer of *ALS3* was 5′-ACGACAAGGTGTACGAATTAACATCT-3′. The upstream primer of the internal gene (*ACT1*) was 5′-TGGGCCAAAAGGATTCTTATG-3′. The downstream primer of the internal gene was 5′-AGATCTTTTCCATATCATCCCAG-3′. The housekeeping gene fragments of *C. albicans* consisted of *AAT1A, ACC1, ADP1, MPIB, SYA1, VPS13*, and *ZWF1B* (Bougnoux et al., 2003). Quantitative expression of *ALS3* levels was conducted by real-time RT-PCR with a LightCycler 96 system (Roche, Switzerland). The amplification consisted of denaturation at 95°C for 30 s (s), annealing at 95°C for 3 s, and extension at 60°C for 30 s (40 cycles). Each reaction was run in triplicate. *ALS3* expression was normalized to *ACT1*. Expression levels of the regulatory gene and *ALS3* were determined using the delta-delta Ct (2^–△△*Ct*^) method. Ct indicates the average threshold period of the genes obtained in three independent experiments (Escribano et al., 2017). Data are presented as mRNA transcripts (arbitrary units) relative to *ACT1* (Hosseini et al., 2019).

### Biofilm Formation Experiment *in vitro*

All 55 strains *C. albicans* were added to 12-well culture plates at 2 mL/well. A 10 mm sterile indwelling catheter was placed in each well. The culture plates were incubated in a 150 rpm shaking table at 35°C for 90 min (min). Then, culture plates and catheters were washed three times using aseptic saline and cultured in YPG medium for 72 h (h) at 37°C. Biofilm formation *in vitro* was completed in the 12-well culture plates (Deng et al., 2016, 2017).

### Biofilm Formation Experiment *in vivo*

Six-week-old male C57 mice weighing 20.24 g ± 1.78 g (*n* = 50, Beijing Vitonlihua Experimental Animal Technology Co., Ltd., Beijing, China) were randomly divided into three groups. On the basis of *ALS3* expression in *C. albicans*, mice were grouped into either the high *ALS3* expression group (20 mice), low *ALS3* expression group (20 mice), or blank control group (10 mice). A 20 mm long sterile indwelling catheter was placed into the abdominal cavity of each mouse. Mice in the two experimental groups received 1 × 10^7^ *C. albicans* with either high or low *ALS3* expression by intraperitoneal injection 48 h later. Mice in the control group did not receive *C. albicans* injection. In all three groups, catheters were kept in the abdominal cavity.

After feeding under the same conditions for 2 weeks, all the three groups of mice were sacrificed by cervical dislocation. Catheters were taken out, washed three times using saline solution, then cultured in YPG medium for 48 h at 37°C. After catheters were cultured, the growing colonies of *C. albicans* were isolated. The randomly amplified polymorphic DNA (RAPD) method (Perrone et al., 2009) was used to determine whether *C. albicans* isolated *in vitro* were the same as those inoculated in abdominal cavities of the mice. Four arbitrary promoters (LEG2, CDL6s, Leptopatho, and CDL6as) were selected for the RAPD method (Vrioni and Matsiota-Bemard, 2001). Finally, *ALS3* expression in *C. albicans* strains was detected, also we performed a drug sensitivity test.

### Prevention of Biofilm Formation in Mice

Six-week-old male C57 mice weighing 21.02 ± 1.12 g (*n* = 40, Beijing Vitonlihua Experimental Animal Technology Co., Ltd, Beijing, China) were divided into four groups in the prevention of biofilm formation experiment. The mice were divided into the voriconazole group, caspofungin group, micafungin group, and control group, with 10 mice in each group. Ten *C. albicans* strains with high *ALS3* expression were selected for the prevention experiment. A 20 mm long sterile indwelling catheter was placed into the abdominal cavity of each mouse. Ten mice in each group received a dose of 1 × 10^7^ *C. albicans* by intraperitoneal injection 48 h later. All mice in the experiment groups received preventive therapy consisting of different antifungal agents, which included voriconazole (10 mg/kg per day), caspofungin (1.5 mg/kg per day), and micafungin (1.5 mg/kg per day) in each group. After feeding under the same conditions for 2 weeks, *C. albicans* were extracted from the *in vitro* cultures.

### Statistics

SPSS 17.0 (SPSS, Inc., Chicago, IL, United States) statistical software was used for statistical analysis. Data were expressed as the mean ± standard error and analyzed by *t*-tests and *F*-tests. Susceptibility tests for antifungal agents were compared using non-parametric tests for independent samples. *P* < 0.05 was considered a statistically significant difference.

## Results

### Biofilm Formation and Morphological Structure *in vitro*

In the biofilm formation experiment, 29 *C. albicans* strains out of the 55 total strains formed biofilms *in vitro*. The *C. albicans* budded and began to form mycelium after 6 h of cell culture *in vitro* under observation with an inverted microscope. *C. albicans* colonies fused with each other and arranged into a network after 12 h of culture. *C. albicans* then clumped along the mycelium formation after 24 h of culture. Finally, the mycelium of *C. albicans* interlaced and formed a membrane network structure inside the entire catheter after 48 h of culture *in vitro*.

### Observation of Ultrastructure Under Transmission Electron Microscope

**FIGURE 1:**
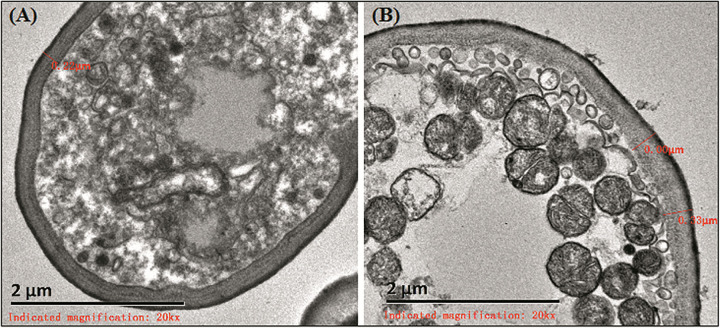
Ultrastructure of biofilms. **(A)** The cell wall of strain that did not form biofilms was about 220 nm. **(B)** The biofilm formation strains were rich in mitochondria.

Cell walls of *C. albicans* were broken in the 26 strains that did not form biofilms. The thickness of most cell walls was about 110–220 nm. Electron density in the strains was higher in structures with spores that sprouted (Figure 1A). In the 29 strains that formed biofilms *in vitro*, cell walls were about 200–350 nm. Electron density in these strains and in structures with spores were the same as those that did not form biofilms. However, the strains that formed biofilms were rich in mitochondria (Figure 1B).

### *ALS3* Expression in the 55 *C. albicans* Strains Prior to the Study

**FIGURE 2:**
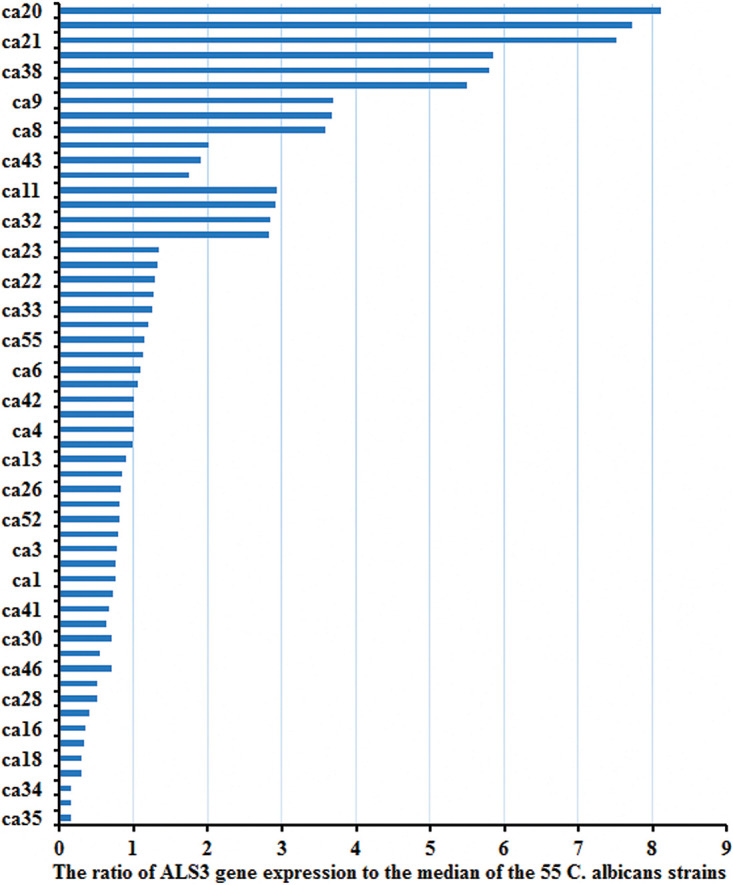
The expression of *ALS3* gene was obvious differences in the 55 strains. There were obvious differences of the *ALS3* gene expression level in the 55 *C. albicans* when they were collected from patients.

There were obvious differences among *ALS3* expression in the 55 *C. albicans* strains after they were collected from patients. The ratio of *ALS3* expression to the median of the 55 *C. albicans* strains is shown in Figure 2.

### Changes in *ALS3* Expression Before and After Culture in all 55 *C. albicans* Strains

**FIGURE 3:**
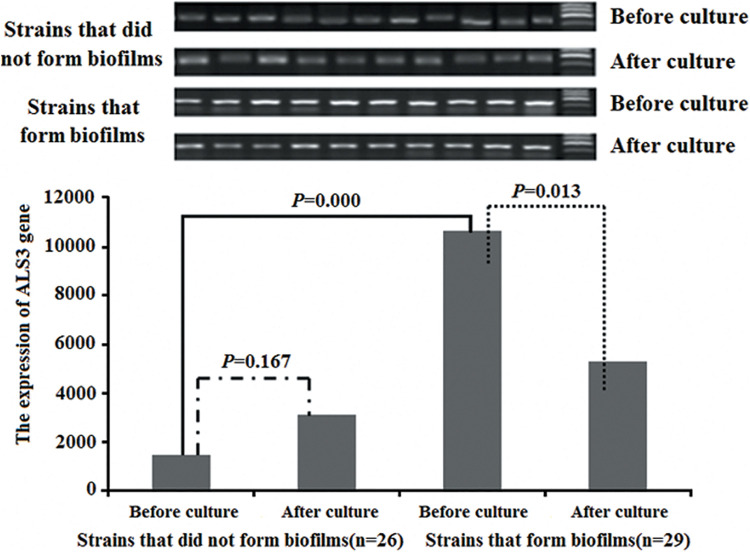
The changes of the *ALS3* gene expression. The average of *ALS3* gene expression was higher in the 29 *C. albicans* with biofilm formation than that of in the 26 *C. albicans* without biofilm formation. The average of *ALS3* gene expression declined after culture in the 29 *C. albicans* with biofilm formation. But there was no different in the 26 *C. albicans* without biofilm formation before and after culture.

Objective stripes were presented at 100–200 bp after electrophoresis of gene amplification products with 2% sepharose gel. The average *ALS3* expression was higher in the 29 *C. albicans* with biofilm formation than that in the 26 *C. albicans* without biofilm formation (*P* = 0.000). The average *ALS3* expression declined after culture *in vitro* in the 29 strains with biofilm formation (*P* = 0.013). However, there were no differences in the 26 strains without biofilm formation before and after culturing (*P* = 0.167; Figure 3).

### Biofilm Formation in Mice

During the biofilm formation experiment in mice, two mice in the high *ALS3* expression group died on the third and fourth days after the sterile indwelling catheter was placed into the abdominal cavity. No mice died in the low *ALS3* expression group or control group. After 2 weeks, the catheters were removed. A total of 14 *C. albicans* strains (14/18, 77.8%) in the high *ALS3* expression group formed biofilms with membrane structures attached to the catheters walls when observed under a microscope.

**FIGURE 4:**
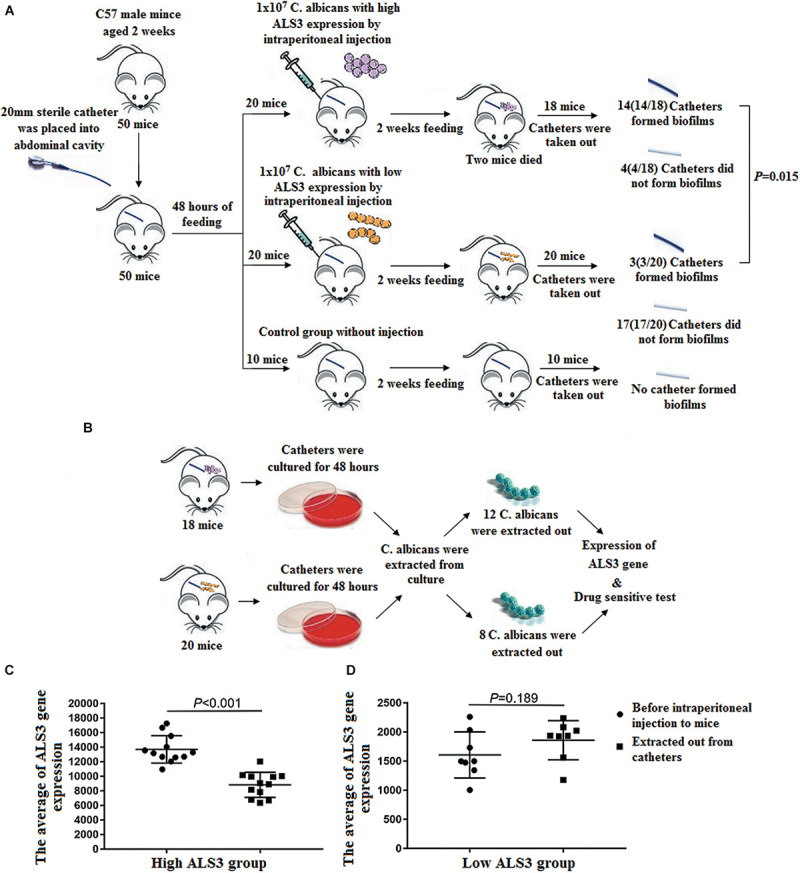
Biofilm formation in mice. **(A)** A total of 14 *C. albicans* (14/18, 77.8%) in the high *ALS3* gene expression group formed biofilms. Three *C. albicans* strains (3/20, 15%) in the low *ALS3* gene expression group formed biofilms (*P* = 0.000). **(B)** After the catheters were cultured, there were 12 *C. albicans* strains from the catheters of the high *ALS3* expression group and the eight *C. albicans* strains from the catheters of the low *ALS3* expression group were isolated from culture *in vitro*. **(C)** The average of *ALS3* gene expression in 12 *C. albicans* from the catheters of the high *ALS3* expression group declined from 13710 ± 1839 to 8828 ± 1680 (*P* < 0.001). **(D)** The average of *ALS3* gene expression in eight *C. albicans* from the catheters of the low ALS3 expression group was 1860 ± 706. While before the biofilm formation experiment, it was 1607 ± 324 in this group (*P* = 0.189).

Three *C. albicans* strains (3/20, 15%) in the low *ALS3* expression group formed biofilms. The rate of biofilm formation in the high *ALS3* expression group was higher than that in the low *ALS3* expression group (*P* = 0.000; Figure 4A). After the catheters were cultured, growing colonies of *C. albicans* were extracted. There were 12 *C. albicans* strains from catheters of the high *ALS3* expression group and eight *C. albicans* strains from catheters of the low *ALS3* expression group that were extracted from culture *in vitro* (Figure 4B). All *C. albicans* strains that were isolated from culture *in vitro* were identified by RAPD as having the same origins as *C. albicans* inoculated in the abdominal cavities of the mice.

### Prevention of Biofilm Formation in Mice

**FIGURE 5:**
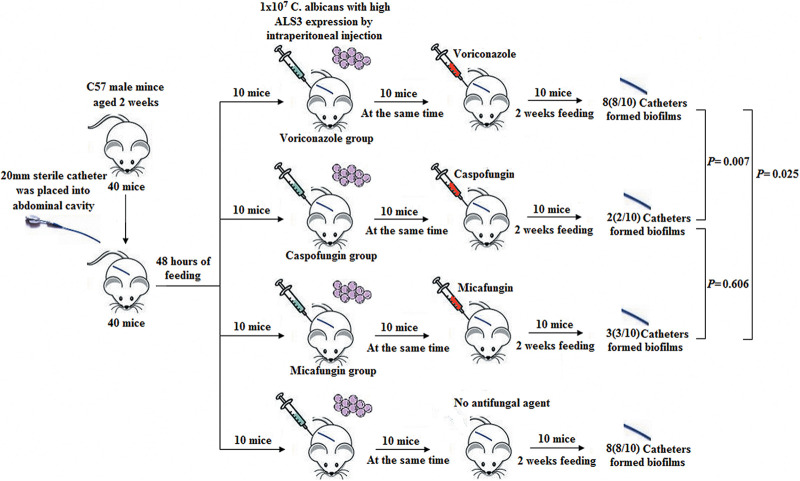
Prevention of biofilm formation in mice. Eight *C. albicans* (8/10, 80%) in voriconazole group, two *C. albicans* (2/10, 20%) in caspofungin group and three *C. albicans* strains (3/10, 30%) in micafungin group formed biofilms with membrane structure attached in the wall of catheters under microscope. The rate of biofilm formation in the caspofungin group and in the micafungin group were lower than that of in the voriconazole group, respectively (*P*_*caspofungin*_ = 0.007 and *P*_*micafungin*_ = 0.025). But there was no different of the biofilm formation rate in the caspofungin and micafungin group (*P* = 0.606).

In the prevention of biofilm formation experiment, eight *C. albicans* strains (8/10, 80%) in the voriconazole group, two *C. albicans* strains (2/10, 20%) in the caspofungin group, and three *C. albicans* strains (3/10, 30%) in the micafungin group formed biofilms with membrane structures attached to the catheter walls when observed under a microscope (Figure 5). The rates of biofilm formation in the caspofungin and micafungin groups were lower than that in voriconazole group (*P*_*caspofungin*_ = 0.007 and *P*_*micafungin*_ = 0.025). However, there were no differences in the biofilm formation rate between the caspofungin and micafungin groups (*P* = 0.606).

### Changes in *ALS3* Expression in *C. albicans* Isolated From Catheters in Mice

After the catheters removed from the mice were cultured *in vitro*, growing colonies of *C. albicans* were isolated. A total of 12 *C. albicans* strains in the high *ALS3* expression group were isolated from the culture system, while eight *C. albicans* strains in the low *ALS3* expression group were isolated. All 12 *C. albicans* strains in the high *ALS3* expression group formed biofilms, while only one *C. albicans* strain formed biofilm in the low *ALS3* expression group. The average *ALS3* expression of 12 *C. albicans* strains in the high *ALS3* gene expression group declined from 13,710 ± 1,839 to 8,828 ± 1,680. The average *ALS3* expression of eight *C. albicans* in the low *ALS3* expression group was 1,860 ± 706, while it was 1,607 ± 324 in the same group before the biofilm formation experiment. There were no differences in average *ALS3* expression before and after the biofilm formation experiment in the low *ALS3* expression group (Figures 4C,D).

### Drug Sensitivity Test *in vitro*

The susceptibility of *C. albicans* to antifungal agents (e.g., fluconazole, voriconazole, itraconazole, caspofungin, and micafungin) *in vitro* was assayed. The activities of antifungal agents against the planktonic form of 55 *C. albicans* strains isolated from blood samples of patients with candidaemia were examined. All antifungal agents showed high antifungal activity against the 55 *C. albicans* strains (Table 1). After the biofilm formation experiment *in vitro*, activities of antifungal agents against the 26 *C. albicans* strains without biofilm formation and 29 *C. albicans* strains with biofilm formation were compared. The *C. albicans* strains with biofilm formation were more resistant to fluconazole, voriconazole, and itraconazole. However, these strains remained sensitive to caspofungin and micafungin. The *C. albicans* strains without biofilm formation were sensitive to all antifungal agents (Table 2).

**TABLE 1:** The number of strains with MIC50 value of different drugs in 55 planktonic *Candida albicans*.

	≤0.06	0.12	0.25	0.5	1	2	4	8	16	32	≥64
Micafungin	89	12	5	2	0	0	0	0	0	NT	NT
Caspofungin	42	51	9	5	1	0	0	0	0	NT	NT
Fluconazole	NT	2	12	21	67	6	0	0	0	0	NT
Itraconazole	NT	1	10	21	62	11	3	0	0	0	NT
Voriconazole	NT	NT	0	0	1	7	23	47	26	4	0

*NT, not tested.*

**TABLE 2:** The number of strains with MIC50 value of different drugs in the 55 *Candida albicans* after biofilm formation experiment *in vitro*.

Concentration	No biofilm					Biofilm				
	Mica	CAS	VOR	ITC	FLU	Mica	CAS	VOR	ITC	FLU
0.06	11	12	NT	NT	NT	1	3	NT	NT	NT
0.12	8	9	0	0	NT	10	14	0	0	NT
0.25	5	4	4	2	0	11	8	0	0	0
0.5	2	1	6	6	0	6	3	2	1	0
1	0	0	11	10	0	1	1	4	5	0
2	0	0	4	6	2	0	0	5	3	0
4	0	0	1	2	6	0	0	11	7	2
8	0	0	0	0	9	0	0	6	10	3
16	0	0	0	0	5	0	0	1	3	4
32	NT	NT	0	0	3	NT	NT	0	1	8
64	NT	NT	NT	NT	1	NT	NT	NT	NT	12
Total	26	26	26	26	26	29	29	29	29	29

After the biofilm formation experiment in mice, activities of antifungal agents against the 12 *C. albicans* strains from the catheters in high *ALS3* expression group and the eight *C. albicans* strains from the catheters in low *ALS3* expression group were compared. The *C. albicans* from the catheters in the low *ALS3* expression group were sensitive to all antifungal agents. The *C. albicans* strains from the catheters in high *ALS3* expression group were more resistant to fluconazole, voriconazole, and itraconazole. These strains remained sensitive to caspofungin and micafungin (Table 3).

**TABLE 3:** The number of strains with MIC50 value of different drugs in the *Candida albicans* after biofilm formation experiment in mice.

Concentration	Low *ALS3* expression group					High *ALS3* expression group				
	Mica	CAS	VOR	ITC	FLU	Mica	CAS	VOR	ITC	FLU
0.06	1	2	NT	NT	NT	1	2	NT	NT	NT
0.12	3	3	0	0	NT	3	5	0	0	NT
0.25	2	2	1	0	0	5	3	0	0	0
0.5	1	1	1	1	0	2	2	0	0	0
1	1	0	1	1	0	1	0	1	0	0
2	0	0	2	1	2	0	0	2	1	0
4	0	0	3	2	3	0	0	3	2	0
8	0	0	1	2	9	0	0	5	6	0
16	0	0	0	0	8	0	0	1	2	1
32	NT	NT	0	0	3	NT	NT	0	1	2
64	NT	NT	NT	NT	1	NT	NT	NT	NT	9
Total	8	8	8	8	8	12	12	12	12	12

## Discussion

*Candida albicans* biofilms are composed of a membranous multifungal complex adsorbed on the surface of biomaterials or a body cavity (Kojic and Darouiche, 2004; Gbelska et al., 2006; Uppuluri et al., 2010). *C. albicans* from biofilms are more invasive and resistant to azole antifungal agents than biofilms originating from planktonic *C. albicans* (Nett et al., 2010; Tobudic et al., 2010; Taff et al., 2013). Central venous catheter therapy is widely used in patients with malignant tumors. When *C. albicans* attaches to the surface of indwells *in vivo* (e.g., in catheters), it is easy to cause catheter-related candidiasis (Høiby et al., 2015). On this basis, *C. albicans* tends to form biofilms (Walraven and Lee, 2013). Biofilm-associated infections are difficult to eradicate because they are a self-perpetuating source of infection and are resistant to a variety of antifungal agents, particularly azole antifungal agents (Taff et al., 2013).

This study selected sensitive *C. albicans* isolated from blood samples from patients with candidiasis. The study intended to verify the results obtained from previous studies that *ALS3* is a predictor of *C. albicans* biofilm formation (Deng et al., 2016, 2017). Further studies plan to provide a theoretical basis for the prevention and treatment of *C. albicans* biofilm formation.

In this study, there was no significant thickening of *C. albicans* cell walls after biofilm formation. This result is different from previous reports (Hamza et al., 2006), in which cell walls after *C. albicans* biofilm formation are twice as thick as those before biofilm formation. However, it was found that after biofilm formation, the electron density and mitochondrial richness of *C. albicans* are high. Another study showed that the sensitivity of *C. albicans* during biofilm formation depends on the metabolic activity of *C. albicans* (Marcos-Zambrano et al., 2014). *C. albicans* biofilm with low metabolic activity was more sensitive to micafungin than that with high metabolic activity. In this study, the electron density and mitochondrial richness of *C. albicans* may have been related to the increased drug resistance after biofilm formation.

It has been reported (Tawara et al., 2000) that *C. albicans* is resistant to fluconazole, voriconazole, and itraconazole after biofilm formation. Although the MIC50 value of caspofungin and micafungin increased, *C. albicans* after biofilm formation remained sensitive to these two antifungal agents. Caspofungin and micafungin overcame the resistance of *C. albicans* after biofilm formation, which is consistent with prior results.

Independent predictors of biofilm formation candida bloodstream infections (CBSIs) were the presence of central venous catheters (CVCs) and urinary catheters (Tumbarello et al., 2012). However, not all *C. albicans* bloodstream infections form biofilms. This study showed that 29 *C. albicans* strains out of the 55 total strains (52.7%) formed biofilms *in vitro*. This result indicates that *C. albicans* had the heterogeneity of biofilm formation. A future goal is to find out the characteristics of *C. albicans* that can form biofilms easily.

The detection and analysis of gene expression is an important factor related to the study of biofilm formation (Sherry et al., 2014). ALS is the main gene family that controls the adhesion of biofilms (Klotz et al., 2007). It has been reported that the expression of *ALS3* is related to biofilm formation and that its expression gradually decreases after biofilm formation (Liu and Filler, 2011). In this experiment, the same results were obtained. This raised the issue of whether there is any difference in the *ALS3* expression before the biofilm formation of *C. albicans*, and whether this difference in *ALS3* expression can predict *C. albicans* biofilm formation? This study found a difference in the expression of *ALS3* in free, antifungal, drug-sensitive *C. albicans*. *In vitro* results showed that the average *ALS3* expression was higher in the 29 *C. albicans* strains with biofilm formation than that in the 26 *C. albicans* strains without biofilm formation. In the following experiments on biofilm formation in mice, similar results were obtained. The rate of biofilm formation was higher in the high *ALS3* expression group than that in the low *ALS3* expression group.

On the other hand, high expression of *ALS3* in *C. albicans* plays an important role not only in biofilm formation, but also in pathogenicity (Mukherjee et al., 2005). This study found that the *C. albicans* strains with biofilm formation were more resistant to fluconazole, voriconazole, and itraconazole. These strains remained sensitive to caspofungin and micafungin. *In vivo* tests in mice showed that the activities of antifungal agents against the 12 *C. albicans* strains from catheters in the high *ALS3* expression group were more resistant to fluconazole, voriconazole, and itraconazole than that in the eight strains from catheters in the low *ALS3* expression group. However, these strains remained sensitive to caspofungin and micafungin. This result regarding the activities of antifungal agents against *C. albicans* with biofilm formation is consistent with previous reports (Jacobson et al., 2009; Simitsopoulou et al., 2013).

It has been shown that the expression of *ALS3* is not significantly different among standard *C. albicans* strains, but there is a significant difference among clinical strains (Bruder-Nascimento et al., 2014), which was consistent with the results in this study. The *C. albicans* strains with high *ALS3* expression were more likely to form biofilms *in vitro* and in mice, leading to increased resistance to azole antifungal drugs but maintenance of sensitivity to caspofungin and micafungin. In the further prevention experiments on biofilm formation in mice, only caspofungin and micafungin prevented *C. albicans* biofilm formation.

Whether the differential expression of *ALS3* in *C. albicans* is a predictor of biofilm formation and whether its expression can further guide the selection of clinical antifungal agents remain to be further explored.

## Conclusion

*ALS3* is differentially expressed in *C. albicans* and *C. albicans* strains with high *ALS3* expression are associated with biofilm formation *in vitro* and in mice. *C. albicans* strains with biofilm formation remain sensitive to caspofungin and micafungin. In the prevention of the biofilm formation experiment, only caspofungin and micafungin prevented formation biofilm formation.

## Data Availability Statement

The datasets presented in this study can be found in online repositories. The names of the repository/repositories and accession number(s) can be found below: https://www.ncbi.nlm.nih.gov/genbank/, XM_705343.2.

## Ethics Statement

The patient gave their written informed consent in accordance with the Declaration of Helsinki. The patient agreed to the use of his specimens and data for our study. The animal study was reviewed and approved by the Tianjin First Central Hospital Medical Ethics Committee.

## Author Contributions

QD: concept and design. KD and WJ: drafting or revising the manuscript. YJ and JC: acquisition of data. WY and XZ: analysis and interpretation of data. All authors contributed to writing, review, and/or revision of the manuscript. QD, WY, and XZ: study supervision. All the authors listed have made a substantial, direct and intellectual contribution to this work, and approved it for publication.

## Conflict of Interest

The authors declare that the research was conducted in the absence of any commercial or financial relationships that could be construed as a potential conflict of interest.
